# Bioterrorism-Related *Bacillus anthracis* Public Health Research Priorities

**DOI:** 10.3201/eid0810.020407

**Published:** 2002-10

**Authors:** Bradley A. Perkins, David A. Ashford

**Affiliations:** Centers for Disease Control and Prevention, Atlanta, Georgia, USA

**Keywords:** anthrax, *Bacillus anthracis*, public health, research, priority-setting

During the 2001 anthrax bioterrorism investigation, several areas were identified in which additional research may improve public health response. The disciplines and specific expertise required to approach many of these areas are varied and reside in multiple entities in the federal government and elsewhere. To identify, prioritize, and coordinate short-term *Bacillus anthracis* bioterrorism research for public health response, the Centers for Disease Control and Prevention (CDC) convened a meeting in Atlanta on December 10–11, 2001, to obtain input on research priorities and improve coordination with federal partners and other stakeholders. One hundred thirty-two representatives from the Department of Health and Human Services (CDC, Food and Drug Administration, and National Institutes of Health), Environmental Protection Agency, Department of Defense, Department of Energy, Department of Justice, U.S. Postal Service, state health departments, universities, and other organizations participated in the meeting. 

The meeting format consisted of two plenary sessions. In the first plenary session experts provided summaries of key topic areas. Background talks were given on evaluation of *B. anthracis*–containing powders or substances; epidemiologic investigation; environmental assessment; surveillance; diagnosis; treatment; postexposure prophylaxis; and remediation. 

After the first plenary session, participants were divided into eight preassigned working groups, covering the following topics: 1) evaluation of *B. anthracis*–containing powders or substances; 2) epidemiologic investigation; 3) environmental assessment; 4) surveillance; 5) diagnosis; 6) treatment; 7) postexposure prophylaxis; and 8) remediation. During the second plenary session, each group presented interim results to the larger group of participants. In two working group sessions, the topic-specific groups identified their three top research priorities and prepared a brief written report describing proposed activities.

For evaluation of *B. anthracis*–containing powders or substances, the top three priorities were 1) rapid analysis of anthrax-containing powder: particle size distribution and matrix characteristics; 2) measurement of particle reaerosolization of different anthrax powder preparations; and 3) development of an in vitro model for the study of cutaneous anthrax by human cell culture. For epidemiologic investigation, priorities were 1) analysis of individual host risk factors for anthrax infection; 2) exposure reconstruction and risk characterization; and 3) review of unexamined or previously unpublished (potentially classified) animal data related to dose response. For environmental assessment, the top priorities were 1) validation and standardization of sampling and sample analysis techniques; 2) evaluation of risk of disease in contaminated environments; and 3) determination of risk of reaerosolization. For surveillance, priorities were 1) expanded veterinary surveillance and integration with human health information; 2) use of alternative sources of data in the surveillance for bioterrorism-related events; and 3) design and validation of surveillance systems to detect complex contamination or release scenarios. For diagnosis, priorities were 1) identification of the earliest detectable event in the continuum from exposure to anthrax to disease (using animal models); 2) evaluation of antigen-detection assays; and 3) development of a library of *B. anthracis *subtypes. For treatment, priorities were 1) investigation of the role of immune and antitoxin therapies; 2) expanded investigation of antibiotic therapies in animal models; and 3) development of other animal models. For postexposure prophylaxis, priorities were 1) evaluation of adherence, barriers to adherence, and adverse events associated with long-term use of antimicrobial agents; 2) pediatric anthrax vaccine safety and immunogenicity studies; and 3) animal challenge studies to optimize postexposure prophylaxis in humans. For remediation, the top three priorities were 1) evaluation of remediation agents; 2) development of risk-based decision templates for sampling and remediation; and 3) reaerosolization studies and agent- and space-specific scenarios.

This meeting defined a framework and set specific priorities for additional research needed to improve public health response to *B. anthracis*–related bioterrorism. Explanations of why the research was considered important, what the research would be, how it would be carried out, who could do it, and when it could begin are available online from URL: http://www.cdc.gov/ncidod/dbmd/diseaseinfo/files/MeetingReport_BTPriorities_Dec1011.pdf). 

